# *N*-(2-Chloro­phen­yl)-4-methyl­benzene­sulfonamide

**DOI:** 10.1107/S1600536809053756

**Published:** 2009-12-19

**Authors:** B. Thimme Gowda, Sabine Foro, P. G. Nirmala, Hartmut Fuess

**Affiliations:** a Department of Chemistry, Mangalore University, Mangalagangotri 574 199, Mangalore, India; b Institute of Materials Science, Darmstadt University of Technology, Petersenstrasse 23, D-64287 Darmstadt, Germany

## Abstract

The mol­ecule of the title compound, C_13_H_12_ClNO_2_S, is bent at the S atom with a C—SO_2_—NH—C torsion angle of −54.8 (2)°. The dihedral angle between the two aromatic rings is 71.6 (1)°. An intra­molecular N—H⋯Cl hydrogen bond is observed. The crystal structure features inversion-related dimers formed by pairs of N—H⋯O hydrogen bonds.

## Related literature

For the preparation of the title compound, see: Gowda *et al.* (2005 ▶). For our studies of the effects of substituents on the structures of *N*-(ar­yl)-aryl­sulfonamides, see: Gowda *et al.* (2009 ▶); Nirmala *et al.* (2009 ▶). For related structures, see: Gelbrich *et al.* (2007 ▶); Perlovich *et al.* (2006 ▶).
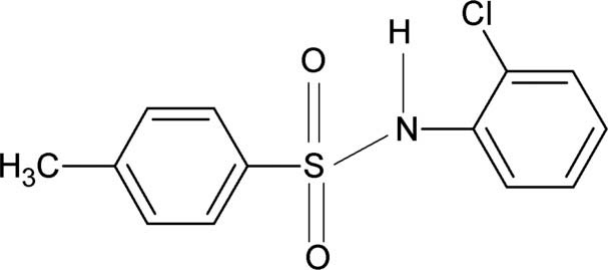


## Experimental

### 

#### Crystal data

C_13_H_12_ClNO_2_S
*M* *_r_* = 281.75
Monoclinic, 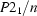

*a* = 8.661 (1) Å
*b* = 9.949 (1) Å
*c* = 15.509 (1) Å
β = 99.384 (8)°
*V* = 1318.5 (2) Å^3^
*Z* = 4
Cu *K*α radiation
μ = 4.00 mm^−1^
*T* = 299 K
0.50 × 0.13 × 0.08 mm

#### Data collection

Enraf–Nonius CAD-4 diffractometer
Absorption correction: ψ scan (North *et al.*, 1968 ▶) *T*_min_ = 0.240, *T*_max_ = 0.741
3151 measured reflections
2351 independent reflections
1792 reflections with *I* > 2σ(*I*)
*R*_int_ = 0.029
3 standard reflections every 3 min
intensity decay: 1.6%

#### Refinement

*R*[*F* ^2^ > 2σ(*F* ^2^)] = 0.039
*wR*(*F* ^2^) = 0.117
*S* = 1.03
2351 reflections
168 parameters
H atoms treated by a mixture of independent and constrained refinement
Δρ_max_ = 0.26 e Å^−3^
Δρ_min_ = −0.32 e Å^−3^

### 

Data collection: *CAD-4-PC* (Enraf–Nonius, 1996 ▶); cell refinement: *CAD-4-PC*; data reduction: *REDU4* (Stoe & Cie, 1987 ▶); program(s) used to solve structure: *SHELXS97* (Sheldrick, 2008 ▶); program(s) used to refine structure: *SHELXL97* (Sheldrick, 2008 ▶); molecular graphics: *PLATON* (Spek, 2009 ▶); software used to prepare material for publication: *SHELXL97*.

## Supplementary Material

Crystal structure: contains datablocks I, global. DOI: 10.1107/S1600536809053756/ci2989sup1.cif

Structure factors: contains datablocks I. DOI: 10.1107/S1600536809053756/ci2989Isup2.hkl

Additional supplementary materials:  crystallographic information; 3D view; checkCIF report

## Figures and Tables

**Table 1:** Hydrogen-bond geometry (Å, °)

*D*—H⋯*A*	*D*—H	H⋯*A*	*D*⋯*A*	*D*—H⋯*A*
N1—H1*N*⋯O2^i^	0.83 (3)	2.40 (3)	3.181 (3)	156 (3)
N1—H1*N*⋯Cl1	0.83 (3)	2.46 (3)	2.952 (2)	119 (2)

Symmetry code: (i) 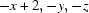.

**Fig. 1.**
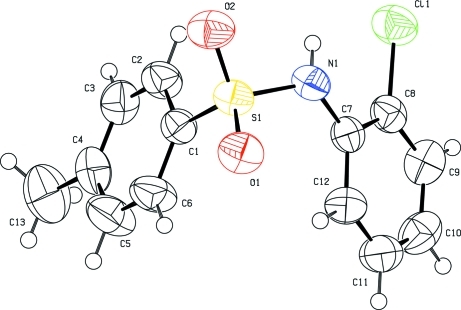
Molecular structure of the title compound, showing the atom labelling scheme. Displacement ellipsoids are drawn at the 50% probability level.

**Fig. 2.**
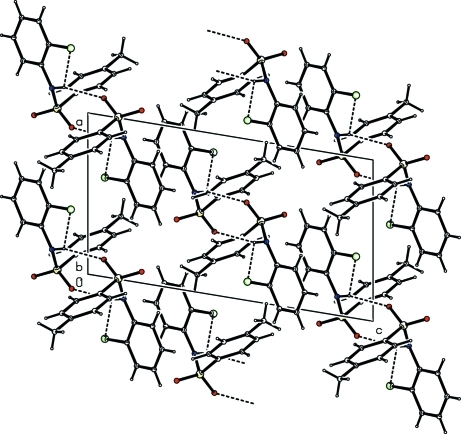
Crystal packing of the title compound. Hydrogen bonds are shown as dashed lines.

## supplementary crystallographic information

### Comment

As part of a study of the effect of substituents on crystal structures of *N*-(aryl)-arylsulfonamides (Gowda *et al.*, 2009; Nirmala *et al.*, 2009), the crystal structure of *N*-(2-chlorophenyl)4-methylbenzenesulfonamide (I) has been determined. The conformation of the N—H bond is *syn* to the 2-chloro group in the aniline benzene ring. The molecule is bent at the S atom with a C1—S1—N1—C7 torsion angle of -54.8 (2)°, compared to the value of -51.6 (3)° in *N*-(phenyl)4-methylbenzenesulfonamide (II) (Gowda *et al.*, 2009) and 60.0 (2)° in *N*-(2-methylphenyl)4-methylbenzenesulfonamide (III) (Nirmala *et al.*, 2009).

The two benzene rings in (I) are tilted relative to each other by 71.6 (1)°, compared to 68.4 (1)° in (II) and 49.7 (1)° in (III). The other bond parameters are similar to those observed in (II), (III) and other aryl sulfonamides (Perlovich *et al.*, 2006; Gelbrich *et al.*, 2007).

The structure exhibits both the intramolecular N—H···Cl and the intermolecular N—H···O(S) hydrogen bonds.

In the crystal structure, pairs of intermolecular N—H···O hydrogen bonds (Table 1) link the molecules into inversion-related dimers. Part of the crystal structure is shown in Fig. 2.

### Experimental

A solution of toluene (10 ml) in chloroform (40 ml) was treated dropwise with chlorosulfonic acid (25 ml) at 273 K. After the initial evolution of hydrogen chloride subsided, the reaction mixture was brought to room temperature and poured into crushed ice in a beaker. The chloroform layer was separated, washed with cold water and allowed to evaporate slowly. The residual benzenesulfonylchloride was treated with 2-chloroaniline in the stoichiometric ratio and boiled for 10 min. The reaction mixture was then cooled to room temperature and added to ice cold water (100 ml). The resultant solid 4-methyl-*N*-(2-chlorophenyl)benzenesulfonamide was filtered under suction and washed thoroughly with cold water. It was then recrystallized to constant melting point from dilute ethanol. The purity of the compound was checked and characterized by recording its IR and NMR spectra. Single crystals used in X-ray diffraction studies were grown by a slow evaporation of an ethanolic solution at room temperature.

### Refinement

The H atom of the NH group was located in a difference map and its positional parameters were refined [N-H = 0.83 (3) Å]. The other atoms were positioned with idealized geometry using a riding model [C-H = 0.93–0.96 Å]. All H atoms were refined with isotropic displacement parameters (set to 1.2 times of the *U*_eq_ of the parent atom).

### Crystal data

**Table:**

C_13_H_12_ClNO_2_S	*F*(000) = 584
*M**_r_* = 281.75	*D*_x_ = 1.419 Mg m^−^^3^
Monoclinic, *P*2_1_/*n*	Cu *K*α radiation, λ = 1.54180 Å
Hall symbol: -P 2yn	Cell parameters from 25 reflections
*a* = 8.661 (1) Å	θ = 6.3–21.3°
*b* = 9.949 (1) Å	µ = 4.00 mm^−^^1^
*c* = 15.509 (1) Å	*T* = 299 K
β = 99.384 (8)°	Needle, colourless
*V* = 1318.5 (2) Å^3^	0.50 × 0.13 × 0.08 mm
*Z* = 4	

### Data collection

**Table:**

Enraf–Nonius CAD-4 diffractometer	1792 reflections with *I* > 2σ(*I*)
Radiation source: fine-focus sealed tube	*R*_int_ = 0.029
graphite	θ_max_ = 67.5°, θ_min_ = 5.3°
ω/2θ scans	*h* = −10→2
Absorption correction: ψ scan (North *et al.*, 1968)	*k* = −11→0
*T*_min_ = 0.240, *T*_max_ = 0.741	*l* = −18→18
3151 measured reflections	3 standard reflections every 120 min
2351 independent reflections	intensity decay: 1.6%

### Refinement

**Table:**

Refinement on *F*^2^	Secondary atom site location: difference Fourier map
Least-squares matrix: full	Hydrogen site location: inferred from neighbouring sites
*R*[*F*^2^ > 2σ(*F*^2^)] = 0.039	H atoms treated by a mixture of independent and constrained refinement
*wR*(*F*^2^) = 0.117	*w* = 1/[σ^2^(*F*_o_^2^) + (0.0678*P*)^2^ + 0.203*P*] where *P* = (*F*_o_^2^ + 2*F*_c_^2^)/3
*S* = 1.03	(Δ/σ)_max_ = 0.003
2351 reflections	Δρ_max_ = 0.26 e Å^−^^3^
168 parameters	Δρ_min_ = −0.32 e Å^−^^3^
0 restraints	Extinction correction: *SHELXL97* (Sheldrick, 2008), Fc^*^=kFc[1+0.001xFc^2^λ^3^/sin(2θ)]^-1/4^
Primary atom site location: structure-invariant direct methods	Extinction coefficient: 0.0074 (7)

### Special details

**Table:**

Geometry. All e.s.d.'s (except the e.s.d. in the dihedral angle between two l.s. planes) are estimated using the full covariance matrix. The cell e.s.d.'s are taken into account individually in the estimation of e.s.d.'s in distances, angles and torsion angles; correlations between e.s.d.'s in cell parameters are only used when they are defined by crystal symmetry. An approximate (isotropic) treatment of cell e.s.d.'s is used for estimating e.s.d.'s involving l.s. planes.
Refinement. Refinement of *F*^2^ against ALL reflections. The weighted *R*-factor *wR* and goodness of fit *S* are based on *F*^2^, conventional *R*-factors *R* are based on *F*, with *F* set to zero for negative *F*^2^. The threshold expression of *F*^2^ > σ(*F*^2^) is used only for calculating *R*-factors(gt) *etc*. and is not relevant to the choice of reflections for refinement. *R*-factors based on *F*^2^ are statistically about twice as large as those based on *F*, and *R*- factors based on ALL data will be even larger.

### Fractional atomic coordinates and isotropic or equivalent isotropic displacement parameters (Å^2^)

**Table:**

	*x*	*y*	*z*	*U*_iso_*/*U*_eq_	
Cl1	0.61691 (9)	−0.13179 (7)	0.05985 (5)	0.0700 (3)	
S1	1.00511 (7)	0.16393 (6)	0.10719 (4)	0.0503 (2)	
O1	1.0698 (2)	0.2235 (2)	0.18850 (12)	0.0636 (5)	
O2	1.1070 (2)	0.0996 (2)	0.05640 (13)	0.0656 (5)	
N1	0.8841 (2)	0.0448 (2)	0.12574 (15)	0.0521 (5)	
H1N	0.867 (3)	−0.009 (3)	0.0838 (19)	0.062*	
C1	0.8918 (3)	0.2840 (2)	0.04327 (15)	0.0471 (6)	
C2	0.8224 (3)	0.2501 (3)	−0.04057 (16)	0.0574 (7)	
H2	0.8376	0.1651	−0.0627	0.069*	
C3	0.7314 (4)	0.3424 (3)	−0.09075 (19)	0.0626 (7)	
H3	0.6840	0.3189	−0.1469	0.075*	
C4	0.7084 (4)	0.4705 (3)	−0.0596 (2)	0.0669 (8)	
C5	0.7801 (4)	0.5015 (3)	0.0238 (2)	0.0827 (10)	
H5	0.7666	0.5868	0.0459	0.099*	
C6	0.8711 (4)	0.4099 (3)	0.07551 (19)	0.0691 (8)	
H6	0.9182	0.4331	0.1318	0.083*	
C7	0.7485 (3)	0.0701 (2)	0.16324 (15)	0.0460 (5)	
C8	0.6155 (3)	−0.0057 (2)	0.13720 (15)	0.0505 (6)	
C9	0.4813 (3)	0.0161 (3)	0.17153 (18)	0.0641 (7)	
H9	0.3933	−0.0368	0.1538	0.077*	
C10	0.4771 (4)	0.1159 (3)	0.23198 (19)	0.0688 (8)	
H10	0.3854	0.1333	0.2539	0.083*	
C11	0.6092 (4)	0.1897 (3)	0.25992 (19)	0.0660 (7)	
H11	0.6073	0.2561	0.3019	0.079*	
C12	0.7438 (4)	0.1671 (3)	0.22683 (17)	0.0597 (7)	
H12	0.8329	0.2173	0.2472	0.072*	
C13	0.6086 (5)	0.5708 (4)	−0.1163 (3)	0.1005 (12)	
H13A	0.5015	0.5415	−0.1254	0.121*	
H13B	0.6440	0.5777	−0.1717	0.121*	
H13C	0.6166	0.6569	−0.0881	0.121*	

### Atomic displacement parameters (Å^2^)

**Table:**

	*U*^11^	*U*^22^	*U*^33^	*U*^12^	*U*^13^	*U*^23^
Cl1	0.0684 (5)	0.0636 (4)	0.0769 (5)	−0.0086 (3)	0.0080 (4)	−0.0160 (3)
S1	0.0432 (3)	0.0513 (4)	0.0539 (4)	−0.0004 (3)	0.0003 (2)	−0.0079 (3)
O1	0.0575 (11)	0.0704 (13)	0.0567 (11)	−0.0045 (9)	−0.0093 (8)	−0.0096 (9)
O2	0.0466 (10)	0.0718 (12)	0.0789 (13)	0.0026 (9)	0.0112 (9)	−0.0164 (11)
N1	0.0515 (11)	0.0439 (12)	0.0604 (13)	0.0025 (9)	0.0078 (10)	−0.0039 (9)
C1	0.0456 (12)	0.0466 (13)	0.0480 (13)	−0.0063 (10)	0.0048 (10)	−0.0050 (10)
C2	0.0664 (17)	0.0524 (15)	0.0499 (14)	−0.0071 (13)	−0.0012 (12)	−0.0088 (11)
C3	0.0664 (17)	0.0697 (18)	0.0486 (14)	−0.0134 (14)	0.0001 (12)	0.0021 (13)
C4	0.0685 (18)	0.0590 (17)	0.0723 (19)	−0.0021 (14)	0.0087 (15)	0.0189 (14)
C5	0.116 (3)	0.0499 (17)	0.076 (2)	0.0135 (18)	−0.0039 (18)	−0.0046 (15)
C6	0.091 (2)	0.0537 (16)	0.0577 (16)	0.0036 (15)	−0.0025 (15)	−0.0152 (13)
C7	0.0493 (13)	0.0429 (12)	0.0446 (12)	0.0071 (10)	0.0038 (10)	0.0078 (10)
C8	0.0556 (14)	0.0453 (13)	0.0479 (13)	0.0042 (11)	0.0003 (11)	0.0054 (11)
C9	0.0512 (15)	0.078 (2)	0.0612 (16)	−0.0008 (14)	0.0050 (12)	0.0063 (15)
C10	0.0616 (17)	0.086 (2)	0.0607 (17)	0.0153 (16)	0.0168 (14)	0.0080 (16)
C11	0.078 (2)	0.0641 (17)	0.0570 (16)	0.0117 (15)	0.0133 (14)	−0.0037 (14)
C12	0.0629 (16)	0.0589 (16)	0.0566 (16)	−0.0002 (13)	0.0076 (13)	−0.0083 (13)
C13	0.107 (3)	0.085 (3)	0.102 (3)	0.011 (2)	−0.005 (2)	0.034 (2)

### Geometric parameters (Å, °)

**Table:**

Cl1—C8	1.737 (3)	C5—H5	0.93
S1—O1	1.4233 (18)	C6—H6	0.93
S1—O2	1.4266 (19)	C7—C8	1.381 (3)
S1—N1	1.639 (2)	C7—C12	1.385 (3)
S1—C1	1.748 (3)	C8—C9	1.373 (4)
N1—C7	1.415 (3)	C9—C10	1.370 (4)
N1—H1N	0.83 (3)	C9—H9	0.93
C1—C6	1.371 (4)	C10—C11	1.369 (4)
C1—C2	1.382 (3)	C10—H10	0.93
C2—C3	1.368 (4)	C11—C12	1.367 (4)
C2—H2	0.93	C11—H11	0.93
C3—C4	1.388 (4)	C12—H12	0.93
C3—H3	0.93	C13—H13A	0.96
C4—C5	1.376 (4)	C13—H13B	0.96
C4—C13	1.506 (4)	C13—H13C	0.96
C5—C6	1.374 (4)		
			
O1—S1—O2	119.12 (12)	C5—C6—H6	120.3
O1—S1—N1	108.46 (12)	C8—C7—C12	118.0 (2)
O2—S1—N1	104.08 (12)	C8—C7—N1	119.4 (2)
O1—S1—C1	108.75 (12)	C12—C7—N1	122.6 (2)
O2—S1—C1	109.52 (12)	C9—C8—C7	121.3 (2)
N1—S1—C1	106.12 (11)	C9—C8—Cl1	118.9 (2)
C7—N1—S1	122.74 (17)	C7—C8—Cl1	119.8 (2)
C7—N1—H1N	112 (2)	C10—C9—C8	119.9 (3)
S1—N1—H1N	111 (2)	C10—C9—H9	120.1
C6—C1—C2	120.2 (3)	C8—C9—H9	120.1
C6—C1—S1	120.8 (2)	C11—C10—C9	119.5 (3)
C2—C1—S1	119.0 (2)	C11—C10—H10	120.3
C3—C2—C1	119.5 (3)	C9—C10—H10	120.3
C3—C2—H2	120.2	C12—C11—C10	120.8 (3)
C1—C2—H2	120.2	C12—C11—H11	119.6
C2—C3—C4	121.4 (3)	C10—C11—H11	119.6
C2—C3—H3	119.3	C11—C12—C7	120.5 (3)
C4—C3—H3	119.3	C11—C12—H12	119.7
C5—C4—C3	117.7 (3)	C7—C12—H12	119.7
C5—C4—C13	121.9 (3)	C4—C13—H13A	109.5
C3—C4—C13	120.4 (3)	C4—C13—H13B	109.5
C6—C5—C4	121.9 (3)	H13A—C13—H13B	109.5
C6—C5—H5	119.1	C4—C13—H13C	109.5
C4—C5—H5	119.1	H13A—C13—H13C	109.5
C1—C6—C5	119.3 (3)	H13B—C13—H13C	109.5
C1—C6—H6	120.3		
			
O1—S1—N1—C7	61.9 (2)	C2—C1—C6—C5	0.4 (4)
O2—S1—N1—C7	−170.36 (19)	S1—C1—C6—C5	−179.4 (3)
C1—S1—N1—C7	−54.8 (2)	C4—C5—C6—C1	0.2 (5)
O1—S1—C1—C6	−3.0 (3)	S1—N1—C7—C8	145.7 (2)
O2—S1—C1—C6	−134.7 (2)	S1—N1—C7—C12	−35.5 (3)
N1—S1—C1—C6	113.5 (2)	C12—C7—C8—C9	1.5 (4)
O1—S1—C1—C2	177.2 (2)	N1—C7—C8—C9	−179.6 (2)
O2—S1—C1—C2	45.5 (2)	C12—C7—C8—Cl1	−178.24 (19)
N1—S1—C1—C2	−66.3 (2)	N1—C7—C8—Cl1	0.6 (3)
C6—C1—C2—C3	−0.8 (4)	C7—C8—C9—C10	0.9 (4)
S1—C1—C2—C3	179.0 (2)	Cl1—C8—C9—C10	−179.4 (2)
C1—C2—C3—C4	0.7 (4)	C8—C9—C10—C11	−2.4 (4)
C2—C3—C4—C5	−0.2 (5)	C9—C10—C11—C12	1.5 (5)
C2—C3—C4—C13	179.6 (3)	C10—C11—C12—C7	1.0 (4)
C3—C4—C5—C6	−0.3 (5)	C8—C7—C12—C11	−2.5 (4)
C13—C4—C5—C6	180.0 (3)	N1—C7—C12—C11	178.7 (2)

### Hydrogen-bond geometry (Å, °)

**Table:**

*D*—H···*A*	*D*—H	H···*A*	*D*···*A*	*D*—H···*A*
N1—H1N···O2^i^	0.83 (3)	2.40 (3)	3.181 (3)	156 (3)
N1—H1N···Cl1	0.83 (3)	2.46 (3)	2.952 (2)	119 (2)

Symmetry codes: (i) −*x*+2, −*y*, −*z*.
